# RILPL2 as a potential biomarker for predicting enhanced T cell infiltration in non-small cell lung cancer

**DOI:** 10.1007/s12026-024-09520-6

**Published:** 2024-07-30

**Authors:** Dongfang Chen, Hongyan Zhang, Lifang Zhao, Xueqing Liu, Yueyan Lou, Peiling Wu, Shan Xue, Handong Jiang

**Affiliations:** Department of Respiratory and Critical Care Medicine, Renji Hospital Shanghai Jiao Tong University School of Medicine, Shanghai, 200127 China

**Keywords:** Non-small cell lung cancer, Rab-interacting lysosomal protein-like 2, T cell infiltration

## Abstract

Our previous bioinformatics analysis has revealed that Rab-interacting lysosomal protein-like 2 (RILPL2) is associated with tumor immune microenvironment in non-small cell lung cancer (NSCLC). In our study, we collected 140 patients with primary NSCLC to verify the RILPL2 expression and its prognostic value, the relationship between RILPL2 expression and CD4^+^, CD8^+^T cell infiltration. A total of 140 patients who had been diagnosed with primary NSCLC (including 66 lung adenocarcinomas and 74 lung squamous cell carcinomas) were enrolled in our study. Immunohistochemical (IHC) staining was performed to analyze the expression of RILPL2, CD4, and CD8 in these patients. Compared with peri-cancer tissues, the RILPL2 expression in NSCLC tissues was significantly lower (*P* < 0.0001). RILPL2 expression was significantly related to clinical stage (*P* = 0.019), and low RILPL2 expression indicated higher stage. Low RILPL2 expression predicted worse overall survival (OS) in NSCLC patients (*P* = 0.017). Correlational analyses revealed that RILPL2 expression was significantly positively correlated with CD4^+^T cell infiltration in NSCLC (*R* = 0.294, *P* < 0.001), LUAD subgroup (*R* = 0.256, *P* = 0.038), and LUSC subgroup (*R* = 0.333, *P* = 0.004); RILPL2 expression was also significantly positively correlated with CD8^+^ T cell infiltration in NSCLC (*R* = 0.263, *P* = 0.002), LUAD subgroup (*R* = 0.280, *P* = 0.023), and LUSC subgroup (*R* = 0.250, *P* = 0.031). In conclusion, RILPL2 expression was downregulated in NSCLC; low RILPL2 expression was significantly related to higher stage and worse prognosis; RILPL2 expression was significantly positively correlated with CD4^+^, CD8^+^T cell infiltration.

**Supplementary Information:**

The online version contains supplementary material available at 10.1007/s12026-024-09520-6.

## Introduction

Lung cancer, with its staggering global morbidity and mortality rates, represents an oncological challenge of unparalleled proportions [1]. Dominating the landscape of lung malignancies, NSCLC commands a formidable presence, encompassing an overwhelming 85% of reported cases, and adds to this daunting scenario. Within the dynamic landscape of cancer research, immunotherapy has emerged as a focal point, reshaping the treatment paradigm for NSCLC [2]. In this era of transformative advancements, the forefront of advanced NSCLC treatment now revolves around the strategic deployment of immune checkpoint inhibitors (ICI_s_) specifically engineered to engage programmed death-1 (PD-1) or its counterpart, programmed death ligand 1 (PD-L1), and these ICI_s_ constitute the cornerstone of the standard first-line approach [3]. This evolution marks a seismic shift in therapeutic strategies, underscoring the pivotal role of immunotherapy in redefining the contours of NSCLC management on a global scale. Despite its success, a subset of patients remain unresponsive to immunotherapy; in addition, most patients inevitably develop resistance to immunotherapy [4]. Current studies have revealed that the ICI-mediated anticancer response depends on PD-L1 expression in tumor cells and the penetration of T cells endowed with the ability to identify and eliminate tumor cells has been documented[5]. T cell subtypes have demonstrated prognostic value in cancer patients and served as indicators of immunotherapy effectiveness [5]. Therefore, the study of T cell infiltration is still a hotspot in the field of cancer research.

In previous studies, Rab-interacting lysosomal protein-like 2 (RILPL2) showed an association with several cellular processes, such as protein transport, vesicle transport, and modulation of lysosomal morphology [6]. In addition, RILPL2 could promote tumor progression via the TUBB3/PTEN pathway in breast cancer, and there were reported associations between RILPL2 and the infiltration patterns of multiple immune cell types in endometrial carcinoma [7, 8]. In addition, our previous bioinformatics analysis confirmed that RILPL2 expression was notably reduced in NSCLC tissues compared to peri-cancer tissues; low RILPL2 expression not only served as a significant prognostic indicator but also correlated with lower infiltration levels of CD4 + and CD8 + T cells [9]. The mechanistic role of RILPL2 in the intricate lesions of real-world NSCLC remains elusive, despite the existence of some examples of scientific studies. During the course of our meticulous investigation, we meticulously collected information about 140 primary NSCLC patients guided by our investigative objectives. Our effort was not only to validate the prognostic significance associated with RILPL2 expression, but also more importantly to unravel the intricate relationship between RILPL2 and the dynamics of CD4 + and CD8 + T cell infiltration. This intricate quest will delve into the unknown effects of RILPL2 in the complex environment of NSCLC in an effort to reveal the ambiguous aspects of its involvement in the immune environment.

## Materials and methods

### Patients

Within the framework of this inquiry, we meticulously integrated a cohort comprising 140 patients (including 66 with lung adenocarcinomas and 74 with lung squamous cell carcinomas), subjected to both histopathological scrutiny and clinical diagnosis, affirming the status of primary NSCLC. Notably, this strategy of combining histopathologic and clinical evaluations strengthened the robustness of our patient cohort, providing two-tiered validation for the precise diagnosis of NSCLC. These patients were identified at Shanghai Jiao Tong University School of Medicine, affiliated with Renji Hospital, spanning the period during the timeframe encompassing January 2008 through December 2013. The study garnered ethical approval from the Ethics Review Committee of Renji Hospital. The course of this study continued until December 2014 when the follow-up activities were completed. The study methodology was rigorously chosen, and all data content followed the pathological classification parameters established by the World Health Organization for lung cancer. The determination of the clinical staging was a key aspect of our analytical framework, which strictly adhered to the scientific regulations set out in the 8th edition of the TNM staging scheme for lung cancer. These strict procedural adjustments highlight the careful organization of our study within an ethical and diagnostic framework that ensures the credibility and precision of the study.

The primary inclusion criteria encompassed: (1) the initial treatment was surgical resection; and (2) the medical records and pathological specimens were complete.

The key exclusion criteria were as follows: (1) diagnosed with small-cell lung cancer or other thoracic cancers; (2) treatment with chemotherapy, radiotherapy, or other therapies before surgical resection; (3) any severe or uncontrolled diseases before surgical resection; (4) Eastern Cooperative Oncology Group Performance Status (ECOG PS) score was not 0–1.

### Immunohistochemical (IHC) staining and analysis

Tissue microarrays were subjected to IHC staining for the examination of RILPL2, CD4, and CD8 expression, employing the standard streptavidin-peroxidase technique as detailed in previous literature [10]. Antibodies against RILPL2 (Thermo Fisher, USA), CD4 (Abcam, Cambridge), and CD8 (Abcam, Cambridge) were utilized at dilutions of 1:200, 1:100, and 1:50. An isotype control consisting of concentration-matched nonspecific rabbit IgG was incorporated. The advanced capabilities of confocal laser scanning microscopes and a range of highly specialized instruments are utilized to visualize and manipulate images and complete relatively complex dissection processes. The perfect combination of these advanced technologies is critical to revealing the subtle complexities embedded in visual data. The confocal laser scanning microscope, with its high-resolution optics, is the primary lens utilized in this study, and is capable of penetrating the microscopic realm with unparalleled precision. This is complemented by a suite of other specialized instruments, each with unique analytical capabilities. Fine-grained image analysis goes beyond the realm of mere observation into the intricate realm of quantitative analysis and spatial relationships. The synergistic integration of cutting-edge technologies further enhances the depth of analysis in this study, ensuring a multifaceted exploration of the visual landscape. Through this comprehensive blend of operational approaches, our study not only visualizes microscopic details, but also harnesses the power of sophisticated analysis to provide a comprehensive narrative that transcends the limitations of traditional imaging techniques.

Two trained pathologists independently assessed the results of IHC staining. In carrying out the necessary quantification of RILPL2 expression by IHC scoring, the complexity involved was defined by a fine calculation of the product of staining intensity and the proportion of positive tumor cells. This integrated scoring approach combines qualitative nuances of staining intensity with quantitative measures of the proportion of tumor cells exhibiting a positive immune response. By employing this multiplicative scoring system, our approach achieves a nuanced assessment that captures the inherent subtleties of RILPL2 expression intensity and distribution in the tumor microenvironment. Specifically, it can be expressed that staining intensity received scores of 0 (negative), 1 (weak), 2 (moderate), or 3 (strong); the percentage of positive tumor cells was categorized with scores of 0 (0%), 1 (0–10%), 2 (10–50%), 3 (50–80%), or 4 (> 80%). Subsequently, RILPL2 expression was dichotomized into low (score ≤ 1) or high (score > 1).

In tumor centers, the density of CD4 + and CD8 + T cell infiltration is shown as “cells/mm^2^.” CD4 + T cell infiltration density was categorized into low-density (≤ 187 cells/mm^2^) and high-density (> 187 cells/mm^2^) groups. Similarly, CD8 + T cell infiltration density was stratified into low-density (≤ 134 cells/mm^2^) and high-density (> 134 cells/mm^2^) groups.

### Correlational analysis

In order to explore the correlation between RILPL2 expression and chemokine gene expression as accurately as possible, our analytical trajectory was realized through the complex landscape of the TISIDB database (http://cis.hku.hk/TISIDB/index.php). This powerful database served as a conduit for us to delve deeper into our research, utilizing its rich resource pool to outline the intricate interconnections between the two aforementioned research objects. With the help of the database’s comprehensive data architecture, this analysis was able to unravel the complex web of molecular relationships and gain a more nuanced understanding of the regulatory dynamics between the two subjects.

### Assessment of the anticancer immunity score

Obtaining gene expression data from the TCGA-LUAD and TCGA-LUSC cohorts is a complex process that requires meticulous categorization into different groups based on the dichotomy of low and high RILPL2 expression. This categorization based on the median value of RILPL2 expression delineates the scope for subsequent in-depth analysis. In order to explore in depth the intricate relationship of RILPL2 expression to this component of the anti-cancer immune score, the rich resources of the TIP database were utilized in this study. The database, which is allowed to be accessed at http://biocc.hrbmu.edu.cn/TIP/index.jsp, is our main portal to a wealth of information. Utilizing its dynamic features, its interface can be fully navigated to reveal the subtle relationships between the subjects under study.

### Statistical analysis

In the arena of statistical examination, we engaged in a comprehensive analysis facilitated by the robust tools of GraphPad Prism 7 and SPSS 22.0 software. This analytical exploration included meticulous profiling of the dataset using the multifaceted capabilities inherent in GraphPad Prism 7. We also utilized SPSS 22.0 software to extract subtle patterns and reveal statistical correlations, thereby emphasizing the depth and precision of our statistical analysis. The presentation of experimental results adhered to expressing the mean value alongside its corresponding standard deviation. Group disparities were assessed through the application of two-tailed Student’s *t*-tests. Survival data underwent meticulous scrutiny through Kaplan–Meier analysis, with group comparisons performed utilizing the log-rank test. Correlations were analyzed by the chi-square test or Spearman correlation. Statistical significance was attributed to *P* values below 0.05.

## Results

### RILPL2 expression and its prognostic value in NSCLC patients

**Fig. 1:**
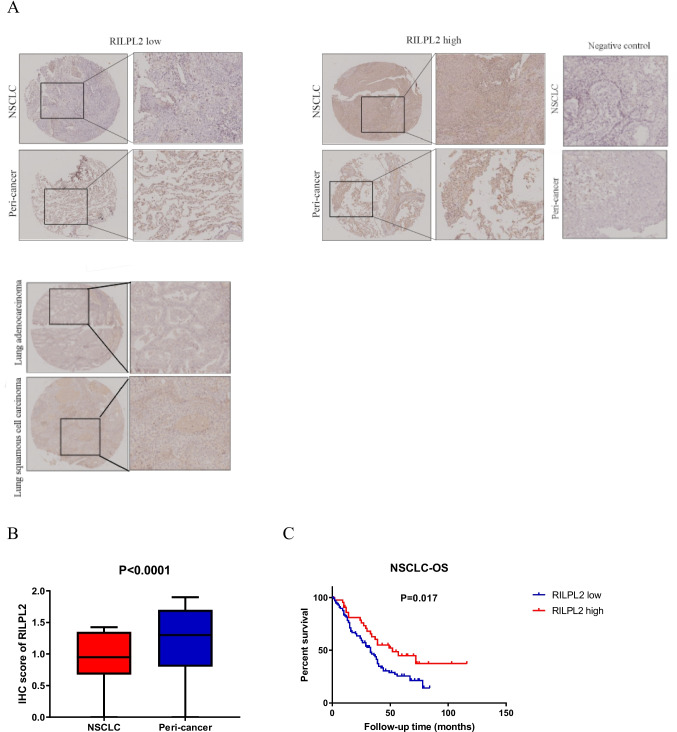
RILPL2 expression and its prognostic value in 140 patients with primary NSCLC. **A** IHC images depicting RILPL2 expression in NSCLC tissues and peri-cancer tissues; IHC images depicting lung adenocarcinoma and squamous cell carcinoma exhibiting identical scoring profiles. **B** The IHC score of RILPL2 in NSCLC tissues and peri-cancer tissues. **C** Kaplan‒Meier survival analysis of patients stratified by RILPL2 expression

We employed IHC staining to validate both the expression and prognostic significance of RILPL2 in a cohort of 140 individuals diagnosed with primary NSCLC. Examination of the IHC images demonstrated the cytoplasmic localization of the RILPL2 protein (Fig. 1A). Compared with that in peri-cancer tissues, RILPL2 expression in NSCLC tissues was significantly lower (*P* < 0.0001) (Fig. 1A and 1B). The results of the K‒M analysis reveal the fact that low RILPL2 expression predicts a poorer OS prognosis among NSCLC patients. This statistically significant association (*P* = 0.017) profoundly reveals the prognostic significance underlying the intricate interplay between RILPL2 expression levels and the overall survival outcomes of the NSCLC patients studied (Fig. 1C).

### The relationship between RILPL2 expression and clinicopathologic features

Following this, our investigative focus pivoted towards an intricate examination of the interplay between RILPL2 expression and a myriad of clinicopathological features within a cohort comprising 140 patients afflicted with primary NSCLC, the final analysis showed that there was a significant correlation between RILPL2 expression and clinical stage (*P* = 0.019) (Table 1), with low RILPL2 expression indicative of a more advanced stage.

**Table 1:** Relationships between RILPL2 expression and clinicopathologic features in 140 patients with primary NSCLC

Variables	No. of patients	RILPL2		*P* value
		Low (*N* = 97)	High (*N* = 43)	
Age(years)				
Median (range)		63(30–84)	62(25–78)	
Gender				
Male	111	75(67.6%)	36(32.4%)	0.389^a^
Female	29	22(75.9%)	7(24.1%)	
Stage				
I–IIIA	114	74(64.9%)	40(35.1%)	0.019^a^
I IIB–IV	26	23(88.5%)	3(11.5%)	
Tumor size (cm)				
≤ 3	32	19(59.4%)	13(40.6%)	0.166^a^
> 3	108	78(72.2%)	30(27.8%)	
Lymph node metastasis				
No	67	47(70.1%)	20(29.9%)	0.832^a^
Yes	73	50(68.5%)	23(31.5%)	

^a^Data were analyzed using the chi-square test

### The relationship between RILPL2 expression and CD4 + and CD8 + T cell infiltration

**Fig. 2:**
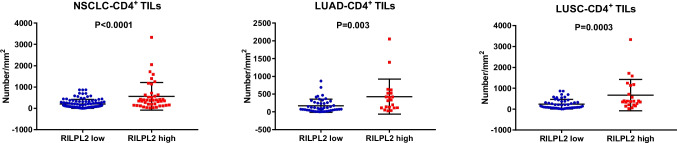
Relationships between RILPL2 expression and CD4 + T cell infiltration in 140 patients with primary NSCLC. **A** IHC images of RILPL2 expression and CD4 + T cell infiltration. **B** The infiltration density of CD4 + T cells in the low RILPL2 expression group and high RILPL2 expression group

**Fig. 3:**
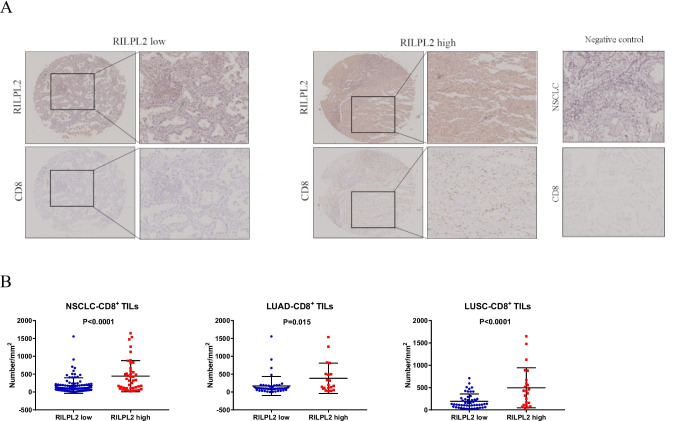
Relationships between RILPL2 expression and CD8 + T cell infiltration in 140 patients with primary NSCLC. **A** IHC images of RILPL2 expression and CD8 + T cell infiltration. **B** The infiltration density of CD8 + T cells in the low RILPL2 expression group and high RILPL2 expression group

IHC staining was employed to validate the expected results in a cohort comprising 140 patients diagnosed with primary NSCLC. The IHC images demonstrate the expression of CD4 and CD8 was obviously greater in the RILPL2-high tumor centers than in the RILPL2-low tumor centers (Figs. 2A and 3A). Correlation analyses revealed that RILPL2 expression demonstrated a significant positive correlation with CD4 + T cell infiltration in NSCLC (*R* = 0.294, *P* < 0.001), LUAD subgroup (*R* = 0.256, *P* = 0.038), and LUSC subgroup (*R* = 0.333, *P* = 0.004) respectively; RILPL2 expression exhibited a significant positive correlation with CD8 + T cell infiltration in NSCLC (*R* = 0.263, *P* = 0.002), LUAD subgroup (*R* = 0.280, *P* = 0.023), and LUSC subgroup (*R* = 0.250, *P* = 0.031) respectively (Tables 2, 3, 4, 5, Supplementary Tables 1, 2). There was a significant increase in the density of CD4 + and CD8 + T cell infiltration in the high RILPL2 expression population, which is a very clear difference (Figs. 2B and 3B). This comparison highlights a substantial difference that reveals an intricate interaction between RILPL2 expression levels and the enhanced immune cell infiltration observed in the high RILPL2 expression group versus the low RILPL2 expression group.

**Table 2:** The correlation between RILPL2 expression and CD4 + T cell infiltration in 140 patients with primary NSCLC

NSCLC samples	CD4		Correlation coefficient	*P* value
	Low	High		
RILPL2 low	58	39	0.294	*P* < 0.001^a^
RILPL2 high	12	31		

^a^Data were analyzed using the chi-square test

**Table 3:** The correlation between RILPL2 expression and CD4 + T cell infiltration in 66 patients with LUAD

LUAD samples	CD4		Correlation coefficient	*P* value
	Low	High		
RILPL2 low	31	15	0.256	0.038^a^
RILPL2 high	8	12		

^a^Data were analyzed using the chi-square test

**Table 4:** The correlation between RILPL2 expression and CD4 + T cell infiltration in 74 patients with LUSC

LUSC samples	CD4		Correlation coefficient	*P* value
	Low	High		
RILPL2 low	27	24	0.333	0.004^a^
RILPL2 high	4	19		

^a^Data were analyzed using the chi-square test

**Table 5:** The correlation between RILPL2 expression and CD8 + T cell infiltration in 140 patients with primary NSCLC

NSCLC samples	CD8		Correlation coefficient	*P* value
	Low	High		
RILPL2 low	57	40	0.263	0.002^a^
RILPL2 high	13	30		

^a^Data were analyzed using the chi-square test

### Correlations between RILPL2 expression and chemokine gene expression

**Fig. 4:**
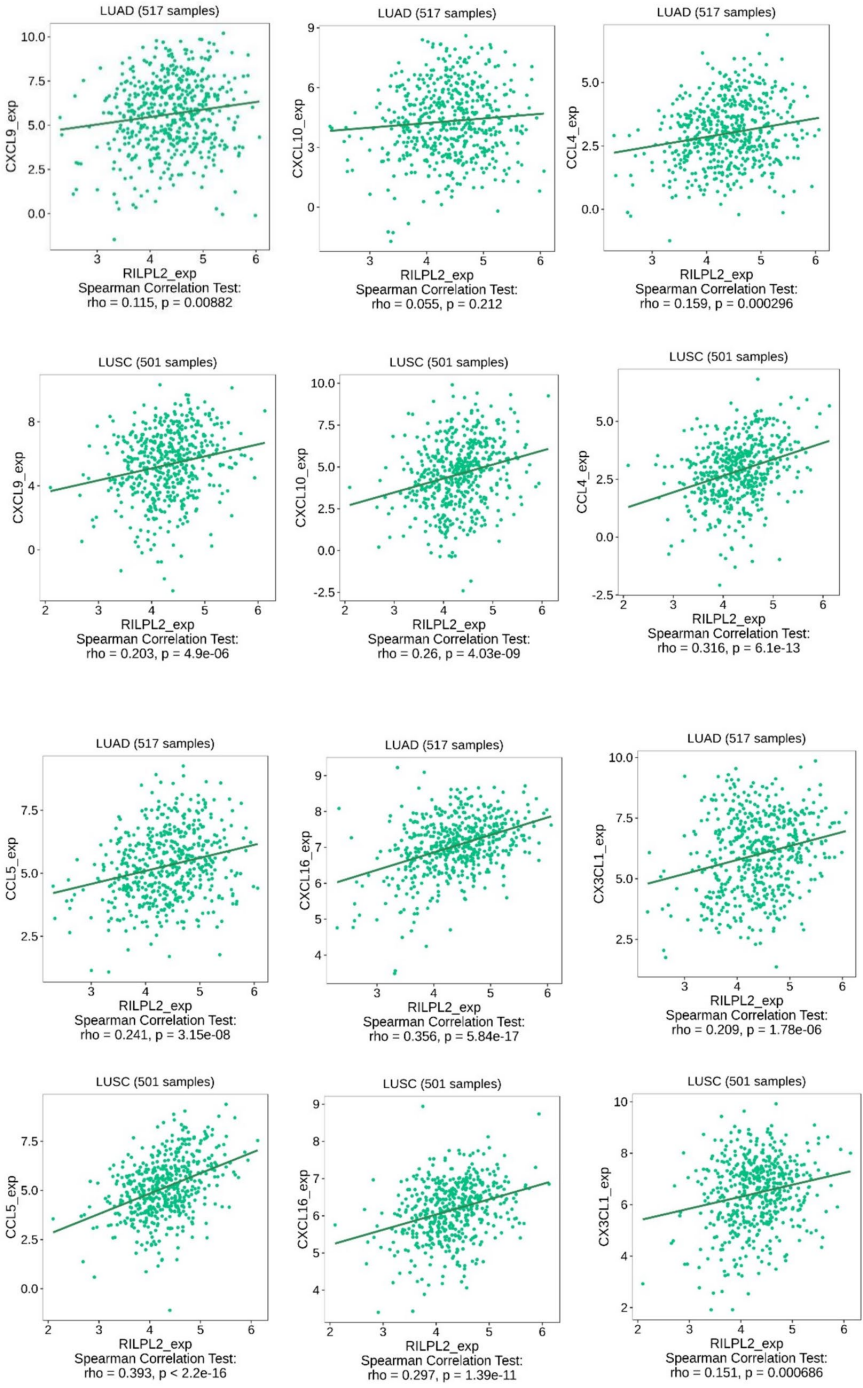
Correlations between RILPL2 expression and chemokine gene expression in NSCLC

By strategically utilizing the TISIDB database, our research was directed towards comprehensively exploring the intricate link between RILPL2 expression levels and the expression profiles of genes exclusively associated with chemokines in the complex environment of NSCLC, which was facilitated by the rich data repository of TISIDB, which not only broadened our understanding of the immune-genome landscape, but also revealed potential avenues for targeted interventions in the intricate pathological network of NSCLC. TISIDB’s rich data repository facilitates this meticulous exploration, which not only broadens our understanding of the immunogenomic landscape, but also reveals potential avenues for targeted interventions in the intricate pathologic network of NSCLC. A comprehensive analysis reveals that within the LUAD, the RILPL2 expression exhibited significant positive correlations with CXCL9 (*R* = 0.115, *P* = 0.00882), CCL4 (*R* = 0.159, *P* = 0.000296), CCL5 (*R* = 0.241, *P* = 3.15e–08), CXCL16 (*R* = 0.356, *P* = 5.84e–17), and CX3CL1 (*R* = 0.209, *P* = 1.78e–06). Within LUSC, a substantial positive correlation was observed between the expression of RILPL2 and CXCL9 (*R* = 0.203, *P* = 4.9e − 06), CXCL10 (*R* = 0.26, *P* = 4.03e − 09), CCL4 (*R* = 0.316, *P* = 6.1e − 13), CCL5 (*R* = 0.393, *P* = 2.2e − 16), CXCL16 (*R* = 0.297, *P* = 1.39e − 11), and CX3CL1 (*R* = 0.151, *P* = 0.000686) expression (Fig. 4).

### The relationship between RILPL2 expression and the anticancer immunity score

**Fig. 5:**
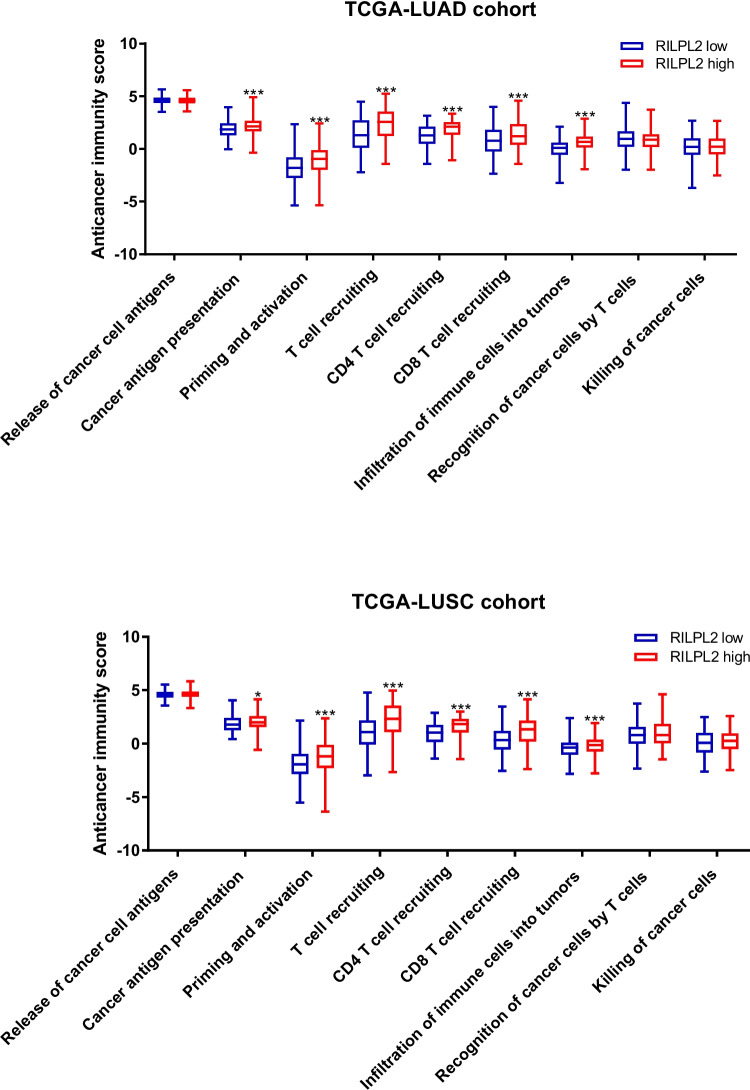
Relationships between RILPL2 expression and anticancer immunity scores based on the TIP database. **A** The total anticancer immunity score in the low RILPL2 expression group and high RILPL2 expression group. **B** Anticancer immunity score across a seven-step cancer-immunity cycle in the low RILPL2 expression group and high RILPL2 expression group

Discussions centered around the TCGA-LUAD and TCGA-LUSC cohorts, and an analysis was performed with the technical support of the TIP database to gain insight into the correlation between RILPL2 expression and anticancer immunity scores. In a comparative analysis against their counterparts in the low RILPL2 expression cohort, the high RILPL2 expression group exhibited significantly elevated scores across multiple aspects of anticancer immunity (Fig. 5).

## Discussion

For the first time, our study validated the prognostic significance of RILPL2 and elucidated the association between RILPL2 expression and CD4 + and CD8 + T cell infiltration within the real-world context of NSCLC. These results align with the outcomes of previous bioinformatics analysis, RILPL2 expression was low in tumor cells in NSCLC, low RILPL2 expression demonstrated a substantial association with advanced disease stage and poor prognosis, and RILPL2 expression exhibited a pronounced positive correlation with CD4 + and CD8 + T cell infiltration.

Tumor-infiltrating lymphocytes (TILs) include T cells, B cells, NK cells, and so on, which are highly heterogeneous lymphocytes that exist within the tumor immune microenvironment, and these factors play a pivotal role in orchestrating the anticancer immune response [11]. CD4 + and CD8 + T cells are the most critical T cells in the tumor immune microenvironment; the main subgroup of CD4 + T cells is CD4 + T helper cells, which can assist B cells in producing antibodies, participating in the activation of CD8 + T cells and activating macrophages to enhance antigen presentation [12]. Cytotoxic CD8 + T cells possess the capability to directly eliminate tumor cells by releasing perforin and granzyme B, mediating apoptosis through the Fas/FasL pathway, or secreting cytokines [13]. In a comprehensive meta-analysis of more than 8600 lung cancer patients, Geng et al. brought forth a profound revelation—an association of heightened levels of infiltrating CD4 + and CD8 + T cells within the very epicenter of tumorous foci with a discernible augmentation in OS[14]. Amidst the research enigma of cancer immunology, the complex study by Liu et al. revealed a marvelous correlation that hints at the potential relevance of RILPL2 in the intricate anticancer immune network, through either direct or indirect means [8]. Our IHC staining results simultaneously highlighted against the finding that there is a significant positive correlation between RILPL2 expression and CD4 + and CD8 + T cell infiltration in NSCLC. This finding goes beyond mere statistical correlations and delves into the intricate molecular dialogue that shapes the immune landscape in the tumor microenvironment. Among the intricacies of the tumor immune process, the travel of effector T cells to the tumor site requires just as much time and efforts for researchers to explore clearly. Complex migratory activities are guided by dynamic interactions between chemokine receptors on effector T cells and their cognate chemokines[15], which is more like being able to determine the spatial navigation of immune effectors, directing them towards the center of the tumor environment. Several chemokines, such as CXCL9, CXCL10, CCL4, CCL5, CXCL16, and CX3CL1, have been identified as drivers of T cell infiltration [16]. For example, high levels of CXCL9 and CXCL10 are associated with increased Th1 cell infiltration; CCL5 promotes the recruitment of CD8 + T cells; heightened CX3CL1 is correlated with augmented TILs [16]. The TISIDB database results indicated a correlation between RILPL2 expression and the mentioned chemokines in NSCLC. We further delved into the relationship between RILPL2 expression and chemokine levels to reveal the mechanism of CD4 + and CD8 + T cell infiltration in NSCLC. Conducting more detailed investigations into the impact of RILPL2 on different T cell subtypes in NSCLC will enhance our comprehension of the specific role played by RILPL2.

With the support of the TIP database, we delved into the relationship between RILPL2 expression and immune scores associated with anticancer response in NSCLC. This sophisticated database, a testament to meticulous computational methodologies, provides a multifaceted lens through which researchers and clinicians can navigate the intricate terrain of immunological landscapes across a diverse spectrum of human cancers [17]. Its inherent capacity to capture the holistic immunophenotypic profiles across such a wide array of malignancies empowers users with a profound understanding of the overarching immune dynamics that transcend cancer type boundaries [17]. The TIP database developers, in a formidable computational endeavor, previously derived the pan-cancer immunophenotype from an expansive dataset comprising 11,373 samples that traverse the landscape of 33 distinct TCGA human cancers [17]. This intricate compilation serves as a comprehensive repository, enabling users to not only conduct efficient analyses but also to visually dissect the dynamic nuances inherent in the realm of anticancer immunity. In addition, the specific infiltration of immune cells can also be observed in seven steps during the cancer immunization cycle [18]. The analysis results of TCGA-LUAD and TCGA-LUSC cohorts revealed that the total anticancer immunity score, cancer antigen presentation score, priming and activation score, T cell (CD4 + and CD8 + T cell) recruiting score, and immune cell infiltration score were significantly higher in high RILPL2 expression groups, which suggested the NSCLC patients expressing high levels of RILPL2 might experience an improved anticancer immune response. These results also suggested that RILPL2 could potentially impact various stages of the cancer-immunity cycle beyond solely influencing immune cell infiltration.

In summary, our investigation substantiates the real-world significance of RILPL2 in NSCLC. Within NSCLC, tumor cells exhibited diminished RILPL2 expression, a factor notably linked to both advanced tumor stage and unfavorable prognosis. Moreover, within our investigative purview, a noteworthy positive correlation materialized, revealing a significant association between the expression levels of RILPL2 and the infiltration dynamics of both CD4 + and CD8 + T cells. In addition, bioinformatics analyses suggested that RILPL2 expression demonstrated a noteworthy positive correlation with various chemokines; high RILPL2 expression indicated a higher anticancer immune score.

## Supplementary Information

Below is the link to the electronic supplementary material.

Supplementary file1 (DOCX 16 KB)

## Data Availability

No datasets were generated or analysed during the current study.
