# Supplementary material for: RILPL2 as a potential biomarker for predicting enhanced T cell infiltration in non-small cell lung cancer
Source: Immunol Res. 2024 Jul 30;72(5):1174–84. doi: 10.1007/s12026-024-09520-6 (PMC11564405; doi:10.1007/s12026-024-09520-6)
Supplement: Supplementary file 1 — Supplementary file1 (DOCX 16 KB) [file 12026_2024_9520_MOESM1_ESM.docx]

Supplementary Table 1 The correlation between RILPL2 expression and CD8^+^T cell infiltration in 66 patients with LUAD

|  | CD8 |  |  |
| --- | --- | --- | --- |
| LUAD samples | Low High | Correlation coefficient | P-value |
| RILPL2 low | 30 16 | 0.280 | 0.023^a^ |
| RILPL2 high | 7 13 |  |  |

a Data were analyzed using Chi-squared test.

Supplementary Table 2The correlation between RILPL2 expression and CD8^+^T cell infiltration in 74 patients with LUSC

|  | CD8 |  |  |
| --- | --- | --- | --- |
| LUSC samples | Low High | Correlation coefficient | P-value |
| RILPL2 low | 27 24 | 0.250 | 0.031^a^ |
| RILPL2 high | 6 17 |  |  |

a Data were analyzed using Chi-squared test.
